# Heat Stress of Algal Partner Hinders Colonization Success and Alters the Algal Cell Surface Glycome in a Cnidarian-Algal Symbiosis

**DOI:** 10.1128/spectrum.01567-22

**Published:** 2022-05-31

**Authors:** Shumpei Maruyama, Paige E. Mandelare-Ruiz, Mark McCauley, Wenjing Peng, Byeong Gwan Cho, Junyao Wang, Yehia Mechref, Sandra Loesgen, Virginia M. Weis

**Affiliations:** a Department of Integrative Biology, Oregon State University, Corvallis, Oregon, USA; b Whitney Laboratory for Marine Bioscience, University of Florida, St. Augustine, Florida, USA; c Department of Chemistry, University of Florida, St. Augustine, Florida, USA; d Department of Chemistry and Biochemistry, Texas Tech University, Lubbock, Texas, USA; University of Thessaly

**Keywords:** algae, climate change, cnidarian, dinoflagellate, endosymbionts, glycoproteins, host-cell interactions, symbiosis

## Abstract

Corals owe their ecological success to their symbiotic relationship with dinoflagellate algae (family Symbiodiniaceae). While the negative effects of heat stress on this symbiosis are well studied, how heat stress affects the onset of symbiosis and symbiont specificity is less explored. In this work, we used the model sea anemone, Exaiptasia diaphana (commonly referred to as Aiptasia), and its native symbiont, Breviolum minutum, to study the effects of heat stress on the colonization of Aiptasia by algae and the algal cell-surface glycome. Heat stress caused a decrease in the colonization of Aiptasia by algae that were not due to confounding variables such as algal motility or oxidative stress. With mass spectrometric analysis and lectin staining, a thermally induced enrichment of glycans previously found to be associated with free-living strains of algae (high-mannoside glycans) and a concomitant reduction in glycans putatively associated with symbiotic strains of algae (galactosylated glycans) were identified. Differential enrichment of specific sialic acid glycans was also identified, although their role in this symbiosis remains unclear. We also discuss the methods used to analyze the cell-surface glycome of algae, evaluate current limitations, and provide suggestions for future work in algal-coral glycobiology. Overall, this study provided insight into how stress may affect the symbiosis between cnidarians and their algal symbionts by altering the glycome of the symbiodinian partner.

**IMPORTANCE** Coral reefs are under threat from global climate change. Their decline is mainly caused by the fragility of their symbiotic relationship with dinoflagellate algae which they rely upon for their ecological success. To better understand coral biology, researchers used the sea anemone, Aiptasia, a model system for the study of coral-algal symbiosis, and characterized how heat stress can alter the algae's ability to communicate to the coral host. This study found that heat stress caused a decline in algal colonization success and impacted the cell surface molecules of the algae such that it became more like that of nonsymbiotic species of algae. This work adds to our understanding of the molecular signals involved in coral-algal symbiosis and how it breaks down during heat stress.

## INTRODUCTION

Coral reefs are among the most vulnerable ecosystems in the current climate crisis (1). As average global temperatures continue to increase, corals are more frequently exposed to their thermal maxima and, as a result, increasingly expel their photosynthetic dinoflagellate partners (family Symbiodiniaceae) in a process known as coral bleaching (1, 2). The loss of their algal symbiont population can leave corals vulnerable to decreased fitness, starvation, disease, and death (1, 2). Heat stress is the primary driver of coral bleaching and lowering the emission of greenhouse gases is the best way to mitigate coral decline globally (3). Nonetheless, a deeper understanding of the molecular mechanisms involved in coral-algal dysbiosis and interpartner signaling is critical in predicting the fate of corals and for the rapid development of solutions that could prolong the survival of coral reefs on a warming planet (3).

While many studies have investigated the effects of heat stress on coral health and coral bleaching, we still lack a clear understanding of how heat stress affects the colonization of hosts by algae and interpartner specificity during the onset of symbiosis. In experiments using the model sea anemone, Exaiptasia diaphana (herein referred to as Aiptasia), heat stress can differentially affect the colonization capacity of symbiodiniaceans native to Aiptasia. Elevated temperatures decrease the colonization capacity of thermally sensitive Breviolum minutum, but not of thermally tolerant B. psygmophilum (4). Heat stress can also cause Aiptasia to preferentially take up thermally tolerant native species of Symbiodiniaceae over sensitive ones (5). Further investigation of how heat affects interpartner signaling mechanisms is critical to understanding how symbiont communities may change within hosts on a warming planet and to predicting the capacity of corals to resist thermal stress.

To date, the potential influence of heat stress on glycan-lectin interactions between algal symbionts and hosts remains unexplored. Glycans are diverse carbohydrates attached to other larger biomolecules such as proteins, lipids, and RNAs (6, 7). There are O- and N-linked glycans in glycoproteins, with O-glycans attached to serine or threonine amino acid residues and N-linked glycans attached to asparagine residues. Glycans have many biological roles, such as aiding in protein folding, cell adhesion, and changing protein function (for review, see reference 7). One important role of glycoconjugates is their function as molecular markers on cell surfaces in host-microbe interactions (7). Cell surface glycans from one partner are recognized by lectins from the other partner, allowing microbes to invade hosts, modulate immunity, or evade host detection altogether (7). In cnidarian-algal symbiosis, molecular manipulation of Symbiodiniaceae glycans or host lectins alters colonization dynamics, suggesting that glycan-lectin interactions are critical for interpartner recognition during the onset of symbiosis (8–13). Glycan lectin interactions may be involved in interpartner specificity because different Symbiodiniaceae species maintain distinct surface glycomes (11, 13, 14). In addition, glycan-lectin signaling may also play a role in maintaining symbiosis because both lectins and glycans are present within the symbiosome even after initial colonization (15–17). Furthermore, host lectin gene expression is influenced by heat stress, suggesting that lectins are associated with dysbiosis (15, 18–23). Finally, heat stress has been found to alter the cell surface proteome in B. psygmophilum, which likely changes its cell surface glycome (24).

Several specific glycan moieties have been characterized in Symbiodiniaceae. High-mannoside glycans are the most abundant type of N-glycan in B. minutum, but increasing the abundance of high-mannoside glycans with inhibitors for glycan maturation pathways can decrease the colonization capacity of B. minutum (12). Similarly, Tortorelli et al. (13) found higher abundances of high-mannoside glycans as measured by lectin array in nonnative species of algae, Cladocopium goreaui, and Fugacium kawagutii, compared to B. minutum, suggesting that an overabundance of high-mannoside glycans can inhibit symbiosis. The second-most abundant group of N-glycans in B. minutum are galactosylated glycans (12). These glycans are less abundant in nonnative species of algae, Symbiodinium pilosum, C. goreaui, and F. kawagutii, as measured by lectin array compared to B. minutum (11, 13). This supports the hypothesis that galactosylation is a marker for a compatible symbiont in Aiptasia (11, 13). Sialylated glycans have also been detected in Symbiodiniaceae in several recent studies, but their role in symbiosis remains unclear, and their abundance is low (11–13). While sialylation has numerous biological roles in the deuterostomes, its presence in other eukaryotic lineages has only recently been acknowledged (25–27). Crucially, genes involved in sialylation have been found in Symbiodiniaceae and cnidarians, including scleractinian corals and Aiptasia, suggesting that sialylated glycans play a role in symbiotic interactions (12, 28).

In this study, we explored how heat stress affected the ability of B. minutum to colonize its native host Aiptasia and its effects on the cell-surface algal N-glycome. We hypothesized that heat stress would hinder the colonization capacity of B. minutum, even when controlling for light stress, algal motility, and cell proliferation. Furthermore, we hypothesized that heat stress would alter the algal glycome in such a way that native algae recognition was hindered.

## RESULTS

### Heat stress hindered the colonization capacity of Breviolum minutum.

**FIG 1:**
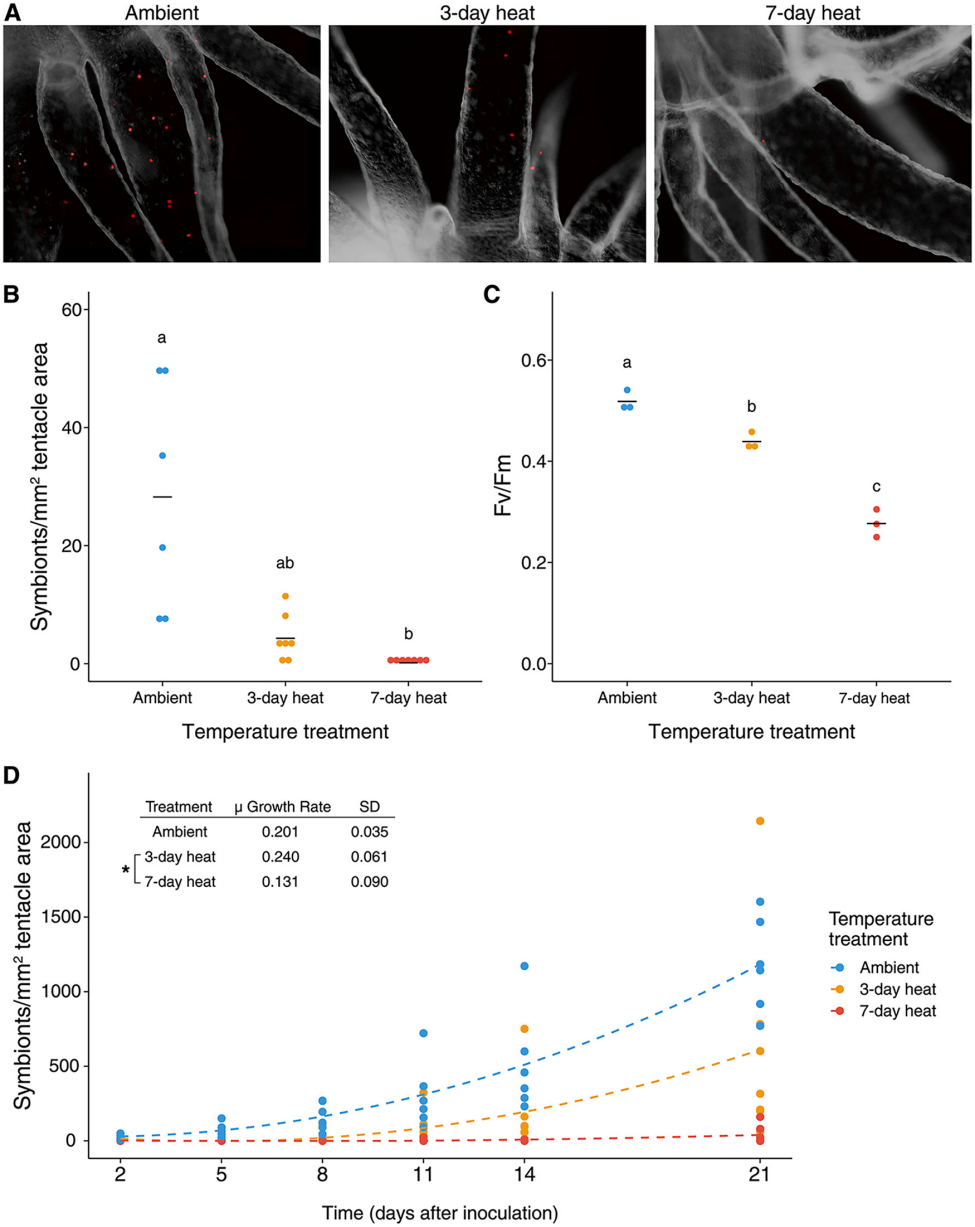
Heat-stressed symbionts have decreased ability to colonize aposymbiotic hosts. (A) Representative images of symbiont densities from each inoculation. (B) Symbiont densities in Aiptasia 2 days after initial inoculation with respective heat-treated algae. (C) Photosynthetic efficiency was measured in heat-stressed algae. (D) Symbiont density was tracked over time over 21 days to determine algal proliferation rates. μ is a unitless slope of the linear regression of the log-transformed data. Letters indicate significant differences (*P* ≤ 0.05) as determined by Kruskal-Wallis and *post hoc* Dunn Tests. Stars indicate significant differences (*P ≤ *0.05) as determined by ANOVA and *post hoc* Tukey test.

Increasing the duration of heat stress reduced the colonization ability of B. minutum (Fig. 1A and B). The same pattern was observed with hourly resuspension of algae and when initial inoculations occurred in darkness (Fig. S4). The maximum quantum yield of photosystem II concomitantly decreased with the increasing duration of heat stress (Fig. 1C). However, as measured by positive staining with Evans blue dye, algal viability was not significantly affected by heat stress (Fig. S5; Kruskal-Wallis test, *P* = 0.10).

Proliferation rates were not significantly different between ambient and 3-day heat-stressed algae (analysis of variance [ANOVA], *post hoc* Tukey HSD, *P* = 0.574). In comparison, proliferation rates in 7-day heat-stressed algae remained significantly lower than in algae from the 3-day heat treatment throughout the experiment (ANOVA, *post hoc* Tukey honestly significant difference [HSD], *P* = 0.02), likely due to near-zero initial colonization densities (Fig. 1D).

### The symbiont glycome changed with heat stress as measured by mass spectrometric analyses and lectin staining.

**FIG 2:**
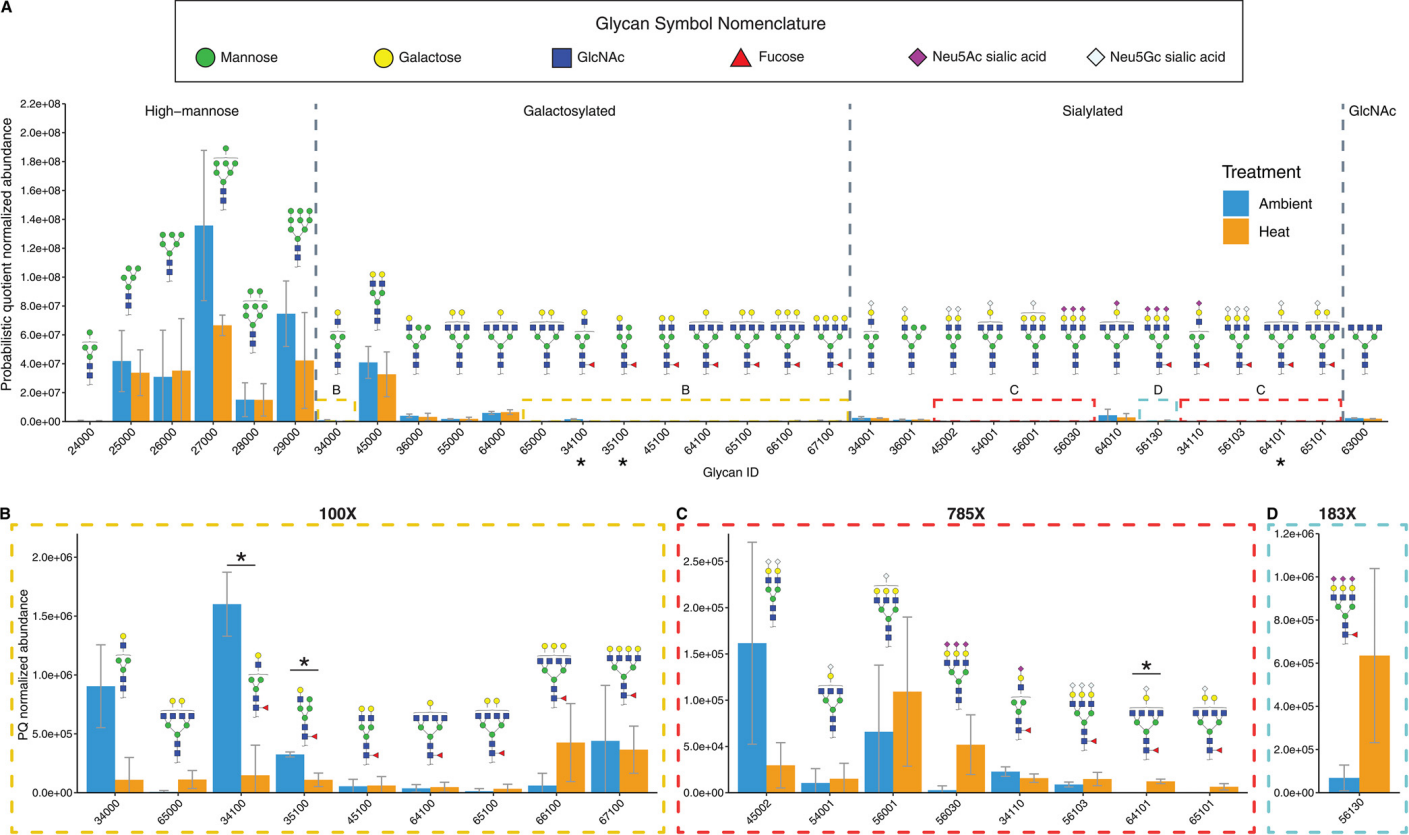
The effect of heat stress on the glycome of Breviolum minutum. (A) Mass spectrometric analysis of PQN normalized glycan abundances of ambient and heat-stressed algae. Glycan structures are represented with illustrations following standards set by the Symbol Nomenclature of Glycans. Glycans are separated into four structural groups by their terminal residues: high mannose, galactosylated, sialylated, and GlcNAc. Boxed sections (B to D) are zoomed-in graphs of corresponding boxes in (A). Statistical differences were determined with FDR adjusted Student’s t-tests. *, *P* < 0.05; *n* = 3.

A total of 32 individual algal N-glycans were characterized from surface glycoproteins by mass spectrometry (Fig. 2A). Three glycans varied significantly in their probabilistic quotient normalization (PQN) abundance between treatments (Fig. 2A). Two galactosylated glycan abundances, IDs 34100 and 35100, were significantly reduced in ambient treatments (Fig. 2B; Student's *t* test false discovery rate [FDR] adjusted, *P* = 0.039, *P* = 0.039, respectively). Glycan IDs 34100 and 35100 were both fucosylated, biantennary hybrid glycans, with a mannose terminal residue on one branch and a galactose terminal residue on the other. Two sialylated glycans, IDs 64101 and 65101, were only identified in heat treatments and were absent from ambient conditions, but only 64101 was significantly enriched (Fig. 2C; Student's *t* test FDR adjusted, *P* = 0.015, *P* = 0.12, respectively). Glycan IDs 64101 and 65101 are tetraantennary glycans with one terminal N-glycolylneuraminic acid (Neu5Gc) and 65101 with an additional terminal galactose residue.

**FIG 3:**
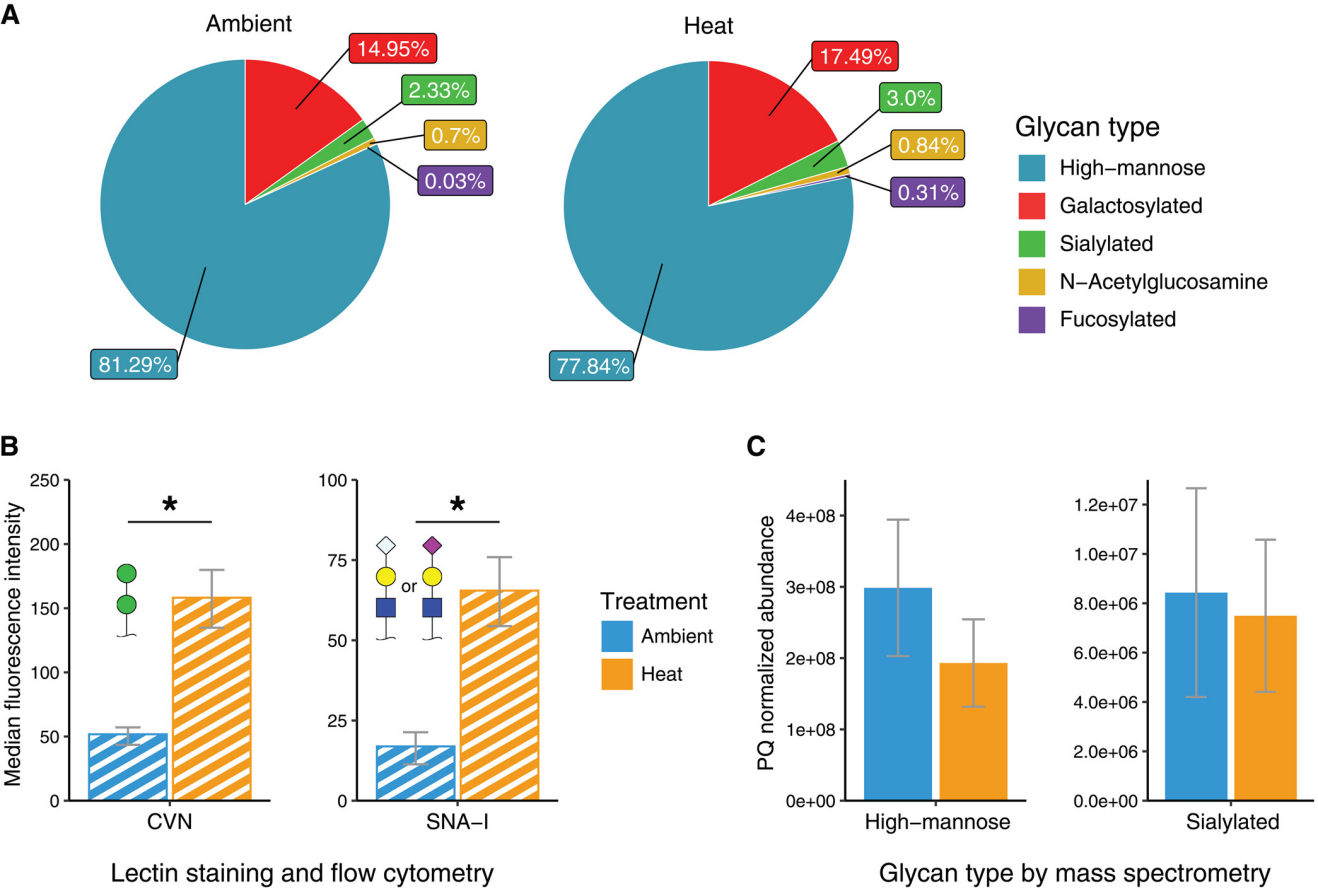
(A) Relative abundance of glycans by type. The “galactosylated” and “sialylated” categories do not include their fucosylated counterparts. Any fucosylated glycans were included in the “fucosylated” category. (B) Median fluorescence intensity of algae stained with phycoerythrin-conjugated lectin. CVN is a high-mannose-specific lectin and SNA-I is a sialic acid-specific lectin. (C) The abundance of glycans was separated by terminal residues as measured by mass spectrometry. The glycan types correspond to their lectin targets in (B).

The N-glycan composition of B. minutum was a largely high-mannose type, contributing an average of 81.3% and 77.8% of all glycans in ambient and heat treatments, respectively (Fig. 3A). The second-largest group was galactosylated glycans, with an average of 15.7% and 18.0% in ambient and heat treatments, respectively (Fig. 2A). A small percentage of glycans were sialylated, with an average of 2.3% and 3.0% in ambient and heat stress treatments, respectively (Fig. 3A). A single oligosaccharide featuring an *N*-acetylglucosamine terminus with an average of 0.7% and 0.8% were identified in ambient and heat treatments, respectively (Fig. 3A). The smallest group were fucosylated glycans (either sialylated or galactosylated), with 0.03% and 0.3% present in ambient and heat treatments, respectively (Fig. 3A).

Lectin staining as measured by flow cytometry revealed that both Cyanovirin-N (CVN) lectin (high-mannoside specific) and Sambucus Nigra lectin (SNA-I; sialic acid-specific) had higher binding in heat-stressed compared to control algae (Fig. 3B and Fig. S6; Student's *t* test, *P* = 0.0014 and *P* = 0.002, respectively), suggesting that both high-mannose and sialic acid glycans were enriched with heat-stress. However, mass spectrometry analysis found that high-mannoside and sialylated N-glycan abundances were lower in heat-stressed algae compared to control algae, although differences were not statistically significant (Fig. 3C; Student's *t* test, *P* = 0.18 and *P* = 0.63, respectively).

## DISCUSSION

### Heat-induced changes in algal physiology affect the colonization capacity of algae.

We found that heat stress hindered the ability of Breviolum minutum to colonize adult Aiptasia (Fig. 1). This corroborates a similar recent study by Kishimoto et al. (4). However, it remains to be determined how heat stress reduces colonization ability (4). Heat stress has been shown to have multiple effects on symbiont physiology, such as decreasing cell viability, division rates, motility, and increasing light-induced generation of reactive oxygen species, all of which influence colonization ability (29–32). The several experiments in this study that were conducted to address each of these confounding variables found that none of these factors alone could explain the observed decline in the colonization of Aiptasia by algae with heat stress.

Overall symbiont density was an order of magnitude higher in inoculations with hourly resuspension of the algae compared to those without resuspensions (Fig. 1B and Fig. S4A). These data suggest that motility and symbiont availability in the water column significantly affects colonization dynamics. This information can inform the design of future colonization experiments. This is particularly important for experiments when algal glycans are masked by exogenous lectins because the addition of certain lectins can halt algal motility (33, 34). In our study, neither equalizing symbiont availability with resuspensions nor conducting inoculations during the 12 h dark period of the light cycle when algae are not motile (35–37), were successful in recovering colonization ability in heat-stressed algae. Therefore, other factors are likely to be contributing to the observed decrease in the colonization of hosts by algae exposed to elevated temperatures.

### The detection method has a significant effect on glycan abundances.

While lectin staining revealed increased high-mannoside and sialylated glycan abundance with heat stress, the mass spectrometric analysis showed an opposing pattern, although differences were not statistically significant (Fig. 3C). The different scopes of detection could explain this discrepancy. The mass spectrometric analysis focused on N-linked glycoproteins, which can be released by PNGase F treatment. The lectin staining approach of intact algal cells is potentially able to detect other glycosylated surface moieties, such as O-linked glycans and glycolipids, in addition to N-glycans (7). Furthermore, PNGase F activity has been shown in plants to be inhibited by α1-3 fucose residues, leading to the incomplete release of N-glycans (38). Even with this potential drawback, PNGase F is a reliable endoglycosidase that cleanly cleaves N-linked glycans from glycoproteins, leaving the deaminated protein and freeing carbohydrates for mass spectrometric analysis (39).

The heating experiment was repeated a second time with smaller sample size, and we cleaved and purified the surface glycans in a simplified PNGase F-based protocol. Using this approach, high-mannoside and sialylated N-glycan abundance increased with heat stress, although not significantly (Fig. S3). In this second analysis, we removed the dialysis and C18 column chromatography purification steps after PNGase F digestion and used the glycan solution directly for permethylation, before mass spectrometric analysis. The fewer cleaning steps increased the number of glycans per sample by an order of magnitude. Molecular analysis of algal surface glycans is still in its infancy. Further refinement of protocols will enable the assessment of the entire surface glycome of symbiotic Symbiodiniaceae.

Below, we compare overall glycan abundance between control and heat-stress algae as measured by lectin staining, and for specific glycan moieties, we discuss differences found in the mass spectrometric data between the two treatment types.

### High mannoside glycan overabundance may hinder symbiosis.

Previous work has suggested that high-mannoside glycans are important for host-symbiont recognition. Work by Tivey et al. (12) found that the colonization capacity of B. minutum declined with increased high-mannoside N-glycan abundance in the Aiptasia symbiosis as measured by CVN lectin staining. In addition, work by Tortotelli et al. (13) found that high-mannoside glycans were consistently more abundant in nonnative algal species, Cladocopium goreaui and Fugacium kawagutii, compared to B. minutum as measured by lectin array. The present study revealed that a heat-induced decrease in colonization capacity of B. minutum correlated with an increase in high-mannoside glycans, as measured by CVN lectin staining. The combined evidence suggests that the overabundance of high-mannoside glycans hinders colonization in the B. minutum-Aiptasia symbiosis. One possible explanation is that the host is mistaking the symbiont for a bacterial pathogen because two coral lectins, Millectin from Acropora millepora and PdC lectin from Pocillopora damicornis, are mannose-specific lectins and have been found to recognize both pathogens and symbionts.

Recently, Tortorelli et al. (13) found that masking host lectins with d-mannose reduced colonization rates of hosts by B. minutum. This could highlight the importance of a certain abundance of high-mannose residues, too high or too low, and colonization rates may decrease. An alternative hypothesis is that adding mannose to host lectins increased the high-mannose lectin signal in hosts, which in turn induces an immune response, leading to the rejection of symbionts. Whatever the cause, their study and ours highlight the importance of mannose in glycan-lectin signaling in the B. minutum-Aiptasia symbiosis.

### Galactosylated glycans may be markers for appropriate symbionts.

The second-most abundant group of glycans in B. minutum were galactosylated glycans (Fig. 3A). We found two (ID 34100 and 35100) that were reduced with heat stress (Fig. 2A). Other studies have suggested that galactosylated glycans have a role in symbiosis. Two galactose-binding lectins SLL-2 and CeCL, from the octocoral Sinularia lochmodes and the coral Ctenactis echinata, respectively, are hypothesized to play a role in symbiosis by inducing motile symbionts to enter a sessile form (16, 34, 40). In the Aiptasia symbiosis, B. minutum has higher galactose abundances compared to nonnative or incompatible species, Symbiodinium pilosum, Cladocopium goreaui, and Fugacium kawagutii (11, 13). Furthermore, the addition of β-d-galactose during inoculation increased the colonization rates of both B. minutum and C. goreaui in Aiptasia (13). Altogether, these data suggest that galactosylated glycans are important for the recognition of suitable partners, and a heat-induced decline in galactosylated glycan causes the host to no longer recognize the symbiont as an appropriate partner.

### Heat-specific sialic acid glycans suggest a complex role for sialylation in symbiosis.

The discovery of sialic acids in Symbiodiniacaeae has been of interest because of their role in host-pathogen interactions in deuterostomes (7, 11–13). Our study provided further evidence that sialic acids are present in Symbiodiniaceae and play a role in symbiosis.

Certain sialic acid moiety abundances increased in response to heat stress. Two sialylated glycans, 64101 and 65101, were only identified in heat treatments and were missing from ambient treatments (Fig. 2C). These glycans are fucosylated, tetraantennary glycans, with a single Neu5Gc sialic acid residue. We hypothesize that they may be produced by a heat-induced error in N-glycosylation pathways, resulting in aberrant sialylation of a tetraantennary glycan such as galactosylated glycan ID 64000 and 65000, lacking the Neu5Gc sialic acid residue. Aberrant sialylation is a hallmark of cancer in humans and can promote tumor metastasis by increasing cell adhesion and migration (41). These aberrantly sialylated glycans are specific to heat-stressed symbionts and may act as a glycan marker for a stressed or inappropriate symbiont for the host.

We found that the binding of sialic-acid-specific lectin SNA-I to algae increased with heat stress (Fig. 3B), and Parkinson et al. (11) found lower SNA-I binding in their lectin array in B. minutum compared to free-living species S. pilosum, suggesting that sialic acids are a marker for inappropriate symbionts. In contrast, lectin array data by Tortorelli et al. (13) found that SNA-I binding was highest in B. minutum and lower in the nonnative species C. goreaui and F. kawagutii. This conflicting evidence suggests a complex role of sialic acids in symbiosis, and specific forms of sialic acids may be more important than others.

Recently discovered glycoRNAs in mammalian cells were found to be heavily sialylated and/or fucosylated, and PNGase F removed the N-glycans off glycoRNAs (6). GlycoRNAs were localized to the cell surface, highlighting their probable role as signaling molecules (6). Sialylated and/or fucosylated glycans were well represented in our data, and glycoRNAs could be present in the Aiptasia-Symbiodiniaceae system. Future work on the role of glycoRNAs in coral-algae symbiosis would add to this discussion.

### Fucosylated glycans and xylosylated glycans are poorly represented.

Despite their hypothesized role in cnidarian-algal symbiosis, fucosylated glycans were not well-represented in this study (13). Similarly, xylose was not detected in the data, despite the presence of xylosyltransferases in the B. minutum genome and their prominent role in plant and algal glycomics (12, 42–44). These glycans may be poorly represented in this study due to the limitations of PNGase F because it is inhibited by N-glycans containing core α1-3 fucose, common plant-specific glycosylation that often co-occurs with xylose (45). Future N-glycan analysis would benefit from using additional glycosidases to fully access the surface glycome, including N-/O-linked glycoproteins, glycolipids, and other glycan conjugates.

### Outstanding questions in Symbiodiniaceae glycobiology.

This study describes the effect of heat stress on symbiont glycan diversity, structure, and abundance and is another step in discovering how glycobiology will play a role in coral survival and symbiosis dynamics on a warming planet. Several outstanding questions remain in the study of glycan-lectin interactions in cnidarian-algal symbiosis:

(i) Does the algal glycome change with heat stress *in hospite,* and does it play a role in coral bleaching? Glycome dynamics of cultured algae are likely not the same as those occurring in algae *in hospite*, and it is unclear if changes in the algal glycome will negatively impact already established symbioses.
(ii) Do other environmental factors affect the algal glycome? Other environmental factors such as nutrition and light play a role in symbiont physiology and may also influence the glycome.
(iii) Do thermally tolerant symbionts have a stable glycome? Thermally tolerant symbionts, such as B. psygmophilum, do not lose colonization ability with heat stress (4). In preliminary experiments, we found that while colonization rates of Durusdinium trenchii in Acropora tenuis larvae were not affected by heat stress, a heat-induced decline in CVN labeling of D. trenchii was observed (Fig. S7). This suggests that high-mannose glycan abundances are not important for the colonization of A. tenuis larvae. Unfortunately, due to pandemic-related travel restrictions, we were unable to explore glycan interactions in coral symbioses further.

The chemical analyses of surface glycoproteins and their carbohydrate composition has emerged as a powerful tool to study cellular interactions on the molecular level. In the field of cnidarian-algal symbiosis, we are beginning to understand what specific glycans are important to symbiosis by chemical manipulation of glycans and by studying species-specific differences in the glycome. Our study found that heat stress can alter the glycome to become more like that of noncompatible symbiont species. We do not provide direct evidence, however, that an altered glycome caused the heat-induced decrease in colonization capacity of B. minutum. This and other glycan studies have been performed in the Aiptasia-B. minutum symbiosis suggests that certain glycans moieties play a role in symbiosis and lays a foundation for our understanding of glycan-lectin interactions in coral-algal symbioses. However, corals and their symbionts very likely have different glycans that promote and hinder symbiosis. Therefore, future work must begin to study these interactions in corals and their algal symbionts and test hypotheses that have arisen from the foundational work conducted with Aiptasia. Finally, with the development of CRISPR-cas9 knockdown techniques in corals (46, 47), it is critical to begin testing the function of host lectins to understand their role in colonization dynamics.

## MATERIALS AND METHODS

### Algal and anemone maintenance.

Cultures of Breviolum minutum (culture ID: Mf1.05b) were grown in silicate-free F/2 media at 25°C. ViparSpectra Timer 165W LED lights (Richmond, CA, USA) were set on a 12h:12h light:dark photoperiod with a light intensity of 55 μmol photons/m^2^/sec. A second incubator for heat-stress treatments was set at 32°C with identical light conditions. Aposymbiotic Aiptasia (strain ID: H2) was initially generated by menthol bleaching and maintained in the dark at 25°C. Animals were fed three times a week *ad libitum* with freshly hatched *Artemia* nauplii. Before inoculation experiments, individual anemones were moved to 6-well plates in 7 mL of artificial filtered seawater (FSW) and starved for 5 days in the light. Before inoculation with symbionts, each anemone was viewed under fluorescence microscopy (Zeiss Axio Observer A1) to confirm the absence of symbiont chlorophyll auto-fluorescence.

### Heat-stress treatments.

For treatments, cultures of B. minutum were prepared in 100 mm diameter plastic petri dishes (VWR; Radnor, PA, USA) at a density of 1 × 10^6^ cells/mL in 25 mL of silicon-free F/2 media and sealed with parafilm. Each petri dish was placed into respective incubators set at either 25°C or 32°C for different durations depending on their treatment: “ambient” treatments were incubated at 25°C for 7 days, “3-day heat” treatments were incubated at 25°C for 4 days then transferred to the 32°C incubators for 3 days, and “7-day heat” treatments were incubated at 32°C for 7 days. Algae generated for glycan and lectin analyses were cultured in 3 mL volumes in 35 mm diameter petri dishes (VWR) for 3 days, in identical conditions as previously described.

### Assessing algal health.

The dark-adapted maximum quantum yield of photosystem II (Fv/Fm) was measured using a custom-built fast repetition rate fluorometer (FRRf) (48). Briefly, algae were adapted to the dark for 30 min in either room temperature for ambient treatments or a 32°C water bath for heat treatments. Excitation was delivered at a wavelength of 475 nm in four distinct phases. During the first phase, a saturating sequence of flashlets, each lasting 0.7 μs with a gap of 1.5 μs for a total of 100 flashlets, was delivered. Then a relaxation phase of 80 flashlets begins with a gap of 20 μs, increasing exponentially until the end of the sequence. The third phase was a sequence of 1 600 flashlets lasting 2 μs each and with a 40 μs gap. The final relaxation phase was identical to the second phase.

Cell viability was tested using Evans blue dye (49). A stock solution of 0.05% wt/vol Evans blue (Sigma-Aldrich; Burlington, MA, USA) dissolved in FSW was diluted in a 1:5 ratio of dye to algal cells. Cells were incubated in the dye for 5 min before quantifying cell viability with a hemocytometer. Cells that took up the blue dye were counted as dead.

### Colonization assays.

Aposymbiotic Aiptasia was randomly assigned to each algal heat treatment (ambient, 3-day heat, 7-day heat). Algae from petri dishes were centrifuged for 5 min at 800 × *g*, and the medium was replaced with FSW to dilute the algae to a density of 5 × 10^5^ cells/mL. To inoculate anemones, seawater was removed from anemone wells and replaced with the respective algal treatment. Animals were immediately fed 40 μL of brine shrimp extract after adding algae to induce a feeding response and facilitate the inoculation of algae. The algae were allowed to colonize the anemones for 24 h at 25°C under 12h:12h light:dark conditions.

Twenty-four hours after initial inoculation, the algal suspension was removed, and the anemones were rinsed with 7 mL of FSW to remove any remaining algae. The animals were then transferred to a new 6-well plate with 7 mL FSW to further minimize the number of algal cells outside the host, which could interfere with subsequent analysis of symbiont density within the host. The inoculated anemones remained in this alga-free seawater for an additional 24 h before imaging.

Heat stress can have multiple effects on the physiology of B. minutum, which could, in turn, affect its ability to colonize Aiptasia. These might include a heat-induced decrease in algal motility and an increase in the generation of reactive oxygen species. To control for the potential effect of heat stress on algal motility, the colonization experiment was repeated with hourly resuspension of the algae by repeated mixing using a 1 mL micropipette during the light period of initial inoculation. To minimize both the generation of reactive oxygen species and algal motility, the colonization experiment was repeated with the initial inoculation taking place for 12 h during the dark period of the light cycle, when algae are not motile (35–37).

### Quantification of symbiont density.

Forty-eight hours after initial inoculation, the Aiptasia were imaged with fluorescence microscopy. Aiptasia was relaxed for at least 10 min with 0.18 M MgCl_2_ dissolved in FSW before imaging. Images of three tentacles per anemone in several focal depths were taken in brightfield and under the red filter set 15 (Carl Zeiss) to visualize algal cell auto-fluorescence and form a z-stack image. Tentacles to be imaged were selected based on their orientation to the horizontal plane. Each manually constructed z-stack was automatically merged into a single image using the ‘auto-blend layers’ function in Adobe Photoshop.

Symbiont density was quantified from the merged photographs using ImageJ. An area of interest within a tentacle was manually selected using the brightfield image, and its 2-dimensional area was quantified in mm^2^. Then the same area of interest was superimposed onto the corresponding auto-fluorescence image, and the number of algal cells within the area was automatically counted based on contrast using the functions ‘watershed’ and ‘analyze particles’. The algal cell counts were normalized to the area of interest, and the symbiont densities of three replicate tentacles were averaged for each anemone.

In the first colonization experiment, each Aiptasia was imaged 2, 5, 8, 11, 14, and 21 days after inoculation. In the subsequent colonization experiments, with hourly resuspension of algae or inoculation in the dark, the Aiptasia were only imaged 2 days postinoculation.

The average growth rate of symbiont density was calculated by plotting the natural log of symbiont density over time and determining the linear regression slope for each anemone over 21 days.

### Flow cytometry and lectin staining.

Lectins Cyanovirin-N (CVN, highly specific to α1-2-mannosides present in high-mannoside oligosaccharides) and Sambucus Nigra lectin (SNA-I, highly specific for α-2,6 sialic acid oligosaccharides attached to a terminal galactose, but also to α-2,3 linkages to a lesser degree) were coupled to phycoerythrin (PE) using the Lightning-Link R-PE Antibody Labeling kit (Novus Biologicals number 703-0010) following the manufacturer’s protocol. Independent samples of ambient and 3-day heat-treated algae were washed with 3.3× PBS, and 2.5 × 10^5^ cells were stained with 5 μg/mL of respective lectins in 200 μL total volume for 2 h in the dark. Two staining replicates were prepared per biological replicate. After staining, algae were washed twice with 3.3× PBS and then resuspended in 1 mL of 3.3× PBS. Samples were then run on a CytoFLEX flow cytometer and data was analyzed using FlowJo software. Unstained ambient and heat-treated algal cells were used to determine gating parameters for corresponding lectin-labeled samples (Fig. S1). First, singlet algal cell populations were identified with forward scatter width and forward scatter area. Then, live algal cells were confirmed by positive chlorophyll autofluorescence signals using 488 nm excitation and detection in the PerCP channel (690/50 bandpass). Median fluorescence intensities (MFI) of the PE signal were obtained from the live algal cell population by excitation at 561 nm and capture in channel PE (585/42 bandpass). Unstained cells were used as blank controls, and the MFI of stained cells was subtracted by the MFI of unstained cells.

### Glycan cleavage and mass spectrometry.

Triplicate samples of B. minutum (4.8 to 6.3 million cells per sample) from independent ambient and 3-day heat temperature treatments were spun down at 3 100 × *g*, decanted, and pellets were frozen at −80°C until further prep. Samples were thawed, washed twice with 1 mL of 2× PBS at 14 000 × *g* for 1 min, washed twice with 1 mL of 1× PBS at 14 000 × *g* for 1 min, and washed with 1 mL MiliQ water at 14 000 × *g* for 1 min, and finally with 1 mL of 50 mM ammonium bicarbonate at 14 000 × *g* for 1 min.

Cells were then treated with glycerol-free PNGase F enzyme (New England Biolabs; Rowley, MA, USA) following the manufacturer’s protocols for nondenaturing reaction conditions to remove N-glycans from cell surfaces. The cells were incubated in 100 μL of Glycobuffer 2, 900 μL ultrapure water, and 3 μL PNGase F (glycerol free) at 37°C for 72 h, with inverting tubes occasionally. A 10K molecular weight cutoff SnakeSkin dialysis tubing (ThermoFisher; Waltham, MA, USA) was prepared by soaking in MiliQ water with constant stirring for 72 h with water changes every 24 h. After PNGase F incubation, each sample was placed into a preprepared dialysis tube and dialyzed in 100 mL ultrapure water at 4°C with constant stirring for 72 h. Every 24 h, the water was replaced with clean ultrapure water, and the removed water (containing glycans) was combined and lyophilized under a high vacuum. The lyophilized dialysates were then dissolved in 5 mL ultrapure water and loaded onto a C18 SPE cartridge to capture glycans. The column was then eluted with 5 mL of 5% acetic acid in water to release glycans, and the sample was lyophilized again.

One milligram from each sample (starting material of N-glycomic analysis) was taken out and desalted by an activated charcoal spin column (Harvard Apparatus). Briefly, the dried sample was dissolved in 85% acetonitrile (MeCN)/water with 0.1% trifluoroacetic acid (TFA). The spin column was washed with 200 μL 85% MeCN/water (with 0.1% TFA) three times, then conditioned with 200 μL 95% MeCN/water (with 0.1% TFA) three times. The sample was loaded on the column, centrifuged, and the pass-through was reloaded twice. The column was then washed with 200 μL 95% MeCN/water (with 0.1% TFA) twice followed by the elution with 200 μL 50% MeCN/water (with 0.1% TFA). Desalted glycans were dried and subjected to reduction and permethylation as described previously (50–52). Briefly, the sample was dissolved in a 10 μL borane-ammonia complex solution (10 mg/mL) and incubated in a 60°C water bath for 1 h. After incubation, 1 mL of methanol was added to the sample and dried out. The addition-drying of methanol was repeated three times to remove borates. Sodium hydroxyl beads (stored in DMSO) were then packed to an empty spin column (Harvard Apparatus) and washed with 200 μL of DMSO twice by centrifuging at 1,800 rpm for 2 min. The reduced sample was dissolved in 30 μL DMSO, 1.2 μL water, and 20 μL iodomethane, and loaded to the column. The column was then incubated at room temperature for 25 min. Next, an additional 15 μL iodomethane was added to the column and incubated for another 15 min. After incubation, permethylated glycan solution was collected by centrifuging at 1,800 rpm for 2 min. The column was then washed with 30 μL of MeCN, and the wash solution was combined with the previous permethylated glycan solution. The combined solution was dried and ready for liquid chromatography-tandem mass spectrometry (LC-MS/MS) analysis.

The LC-MS/MS was performed using an Ultimate 3000 nano-LC system (ThermoFisher Scientific) coupled to an LTQ Orbitrap Velos mass spectrometer (ThermoFisher Scientific). N-glycans derived from 200 μg starting material was injected and separated on a 50 cm C18 micropillar array column (μPAC) with a μPAC trap at 55°C (53). The flow rate was 0.3 μL/min. A gradient was used with mobile phase A (98% water, 2% MeCN, 0.1% formic acid) and mobile phase B (80% MeCN, 20% water, 0.1% formic acid) as follows: 0 to 4 min, 40%B; 4 to 64 min, 40% to 70% B; 64 to 69 min, 70% to 97.5% B; 69 to 89, 97.5% B; 89 to 94 min, 97.5% to 40% B; 94 to 114 min, 40% B. A positive mode was used for MS with a full MS of 60,000 resolution. The data-dependent acquisition was used to select the top 8 most intense ions for collision-induced dissociation tandem mass spectrometry. The normalized collision energy of CID was 35, the activation Q was 0.25, and the activation time was 10 ms. The LC-MS/MS data were first processed by MultiGlycan software, then manually checked using Xcalibur software through full MS and MS^2^ to remove any false-positives. Fig. S2 shows two examples of annotation of high-mannose (Fig. S2A) and complex glycan structures (Fig. S2B), respectively. N-glycan compositions were determined by their masses detected in high-resolution full MS (insets). Then, their structures were further confirmed by matching MS2 fragments to the compositions. According to the general N-glycan biosynthesis pathway (54), N-glycans have a consistent core structure and are synthesized following strict rules in an iterative manner. Therefore, their putative structures could be assigned via full MS and MS2.

The algal heat treatment experiment and glycan mass spectrometric analysis was repeated in 2020. After enzymatic cleavage with PNGase F, the dialysis steps and C18 chromatography were removed from the workflow, and the samples were directly processed for LCMS analysis with similar results (Fig. S3).

Absolute abundance was normalized by probabilistic quotient normalization (PQN) without prior total area normalization, as described by Benedetti et al. (55). PQN is a robust method for normalizing mass spectrometric data and has explicitly proven useful for glycomics (55–57). First, a reference spectrum is generated by calculating the median value of each glycan’s absolute abundance from every sample. A vector of quotients is generated for each sample by dividing the absolute abundance of each glycan by the corresponding value in the reference spectrum. A dilution factor is then calculated for each sample by calculating the median of quotients of a given sample. The final PQN value is obtained by dividing the original abundance value by the dilution factor for each sample. *P* values obtained from Student's *t* test on individual glycan abundances were adjusted by false discovery rate (FDR) analysis.

### Statistical analysis.

All data were tested for normality and homoscedasticity to determine the use of parametric or nonparametric statistical tests.

### Data availability.

The protocol used to analyze symbiont density is publicly available with macros for Adobe Photoshop and ImageJ at dx.doi.org/10.17504/protocols.io.n2bvjb9ngk5w/v2. Raw and PQN glycan data, including statistical analysis, is available as Data Set S3.
