# Supplementary material for: Heat Stress of Algal Partner Hinders Colonization Success and Alters the Algal Cell Surface Glycome in a Cnidarian-Algal Symbiosis
Source: Microbiol Spectr. 2022 May 31;10(3):e01567-22. doi: 10.1128/spectrum.01567-22 (PMC9241721; doi:10.1128/spectrum.01567-22)
Supplement: SUPPLEMENTAL FILE 1 — Supplemental material. Download spectrum.01567-22-s0001.pdf, PDF file, 0.1 MB [file spectrum.01567-22-s0001.pdf]

Supplementary Methods

Inoculation experiments were conducted with *Cladocopium goreaui* (culture ID: LHI-33) and *Durusdinium trenchii* (culture id: D1a Ap2) in *Acropora tenuis* larvae. Algal cultures of each species were kept in sealed 30 mL glass vials in 6 mL of F/2 algal media at a density of 1 x 10^6 cells/mL. Vials containing algae were then placed in a heated water bath, either at 25 °C or 32 °C for 3 days. LED full-spectrum lights were set to 12h:12h light:dark cycle at 30 μmol photons/m^2/s over the water bath.

After heat treatment, cultures were spun down at 1 000 x g for 5 minutes and washed with filtered seawater. Half of the culture was used in inoculation experiments while the other was immediately fixed in 4% paraformaldehyde for later lectin-staining experiments.

Fifteen *Acropora tenuis* larvae 10 days post-fertilization were inoculated with 250 000 cells/mL of respective algal cultures in a 1 mL volume of filtered seawater in wells of a 24 well plate (n=3). Larvae were immediately fed 5 μL of BSE. After 12 hours of inoculation, larvae were rinsed in filtered seawater and fixed in 4% paraformaldehyde. All larvae from each inoculation sample was imaged manually under a fluorescent microscope and the number of algae recorded as alga/larvae.

Paraformaldehyde fixed cultures of *C. goreaui* and *D. trenchii* were washed with 3.3X PBS and 250 000 cells stained with 5 μg/mL of phycoerythrin conjugated CVN lectin in 200 μL total volume for 2 hours in the dark with three staining replicates. After staining, algae were washed twice with 3.3X PBS and then resuspended in 1 mL of 3.3X PBS. Samples were then run on a CytoFLEX flow cytometer and data analyzed using FlowJo software. Algal cell populations were identified with forward and side scatter, and positive chlorophyll autofluorescence signals were confirmed by 488 nm excitation and detection in the PerCP channel (690/50 band pass). Median fluorescence intensities (MFI) of the PE signal were obtained from the total algal cell population by excitation at 561 nm and capture in channel PE (585/42 band pass). Unstained cells were used as blank controls, and the MFI of stained cells were subtracted by the MFI of unstained cells.
