# Supplementary material for: Heat Stress of Algal Partner Hinders Colonization Success and Alters the Algal Cell Surface Glycome in a Cnidarian-Algal Symbiosis
Source: Microbiol Spectr. 2022 May 31;10(3):e01567-22. doi: 10.1128/spectrum.01567-22 (PMC9241721; doi:10.1128/spectrum.01567-22)
Supplement: SUPPLEMENTAL FILE 2 — Supplemental material. Download spectrum.01567-22-s0002.pdf, PDF file, 0.8 MB [file spectrum.01567-22-s0002.pdf]

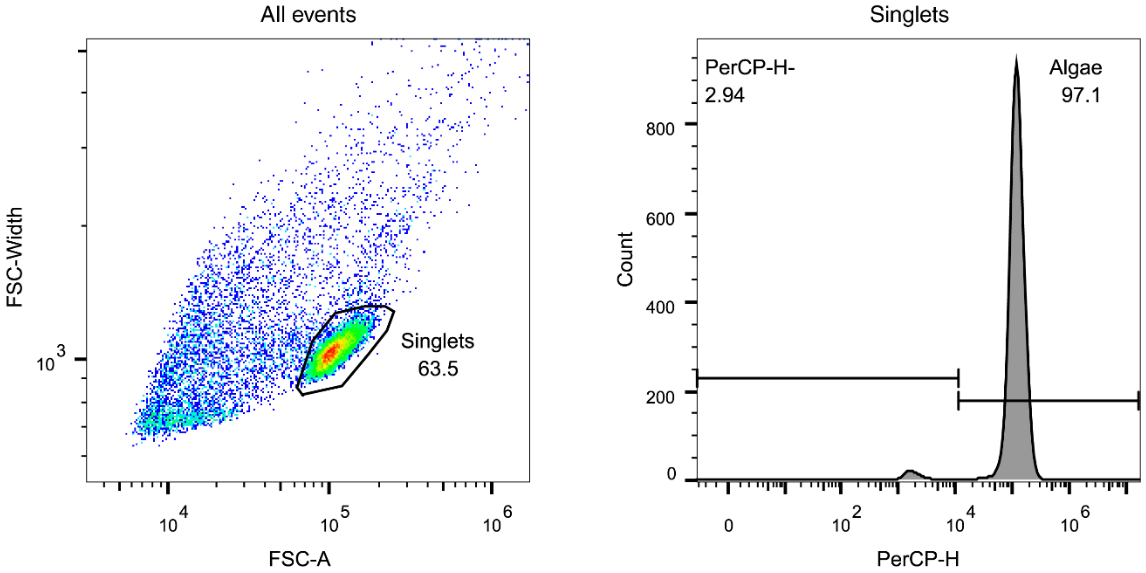

Supplementary Figure 1. Representative cytogram of unstained ambient temperature algal cells. First, singlets were determined by FSC-Width vs FSC-A plots. Then live algal cells were determined using the PerCP channel to detect chlorophyll autofluorescence. Gating strategies were determined using unstained cells and applied to all corresponding lectin-stained samples.

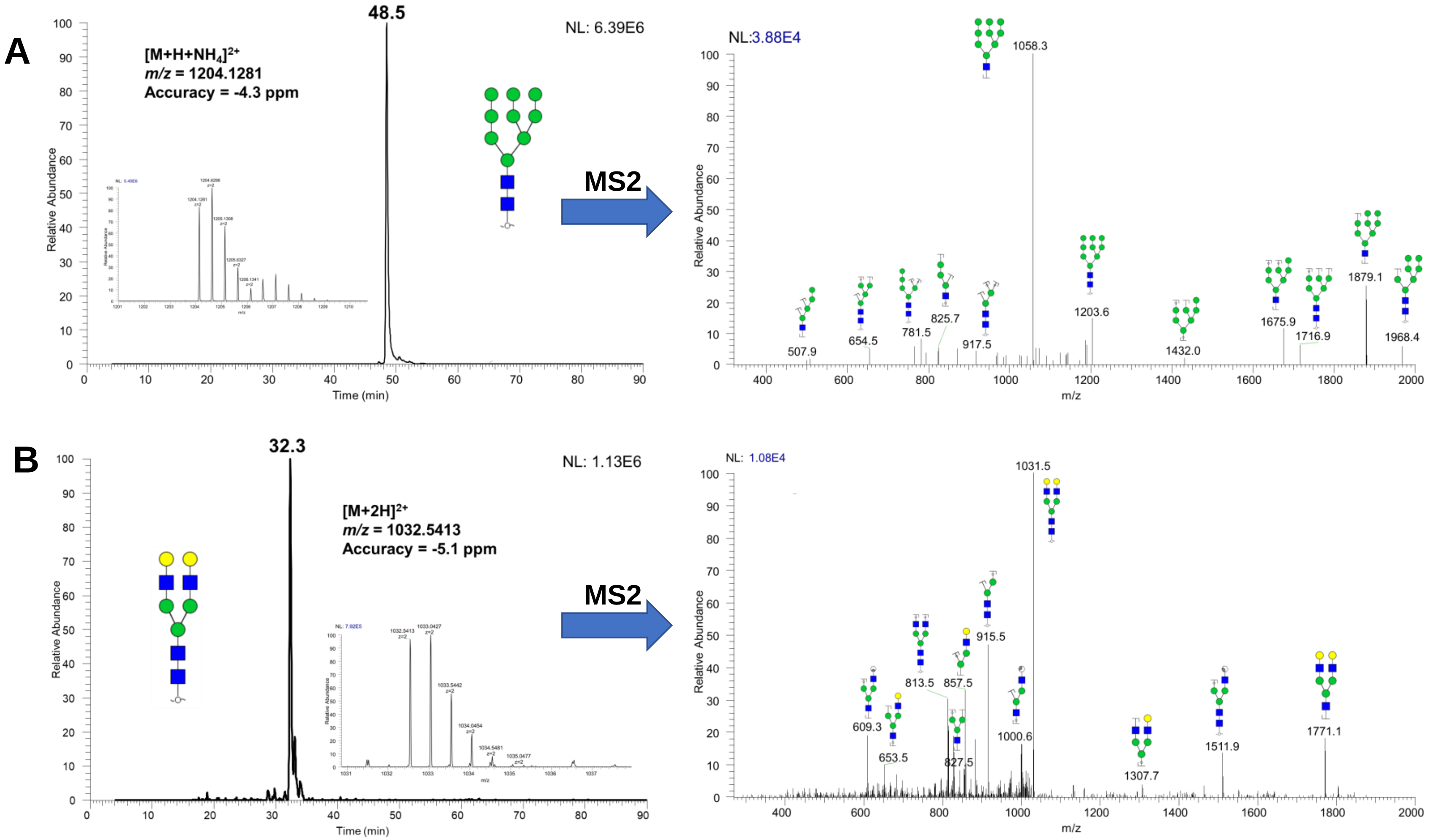

Supplementary Figure 2. Illustration of N-glycan structure annotation for (A) high-mannose and (B) complex glycans, respectively. The insets are corresponding full MS spectra which represent the monoisotopic m/z values and isotopic distributions of the glycans under the LC peak. The fragments in the MS2 spectra can match the putative N-glycan structures, confirming the structural annotation. Symbols: blue square represents N-acetylglucosamine (GlcNAc); green circle represents mannose (Man); yellow circle represents galactose (Gal).

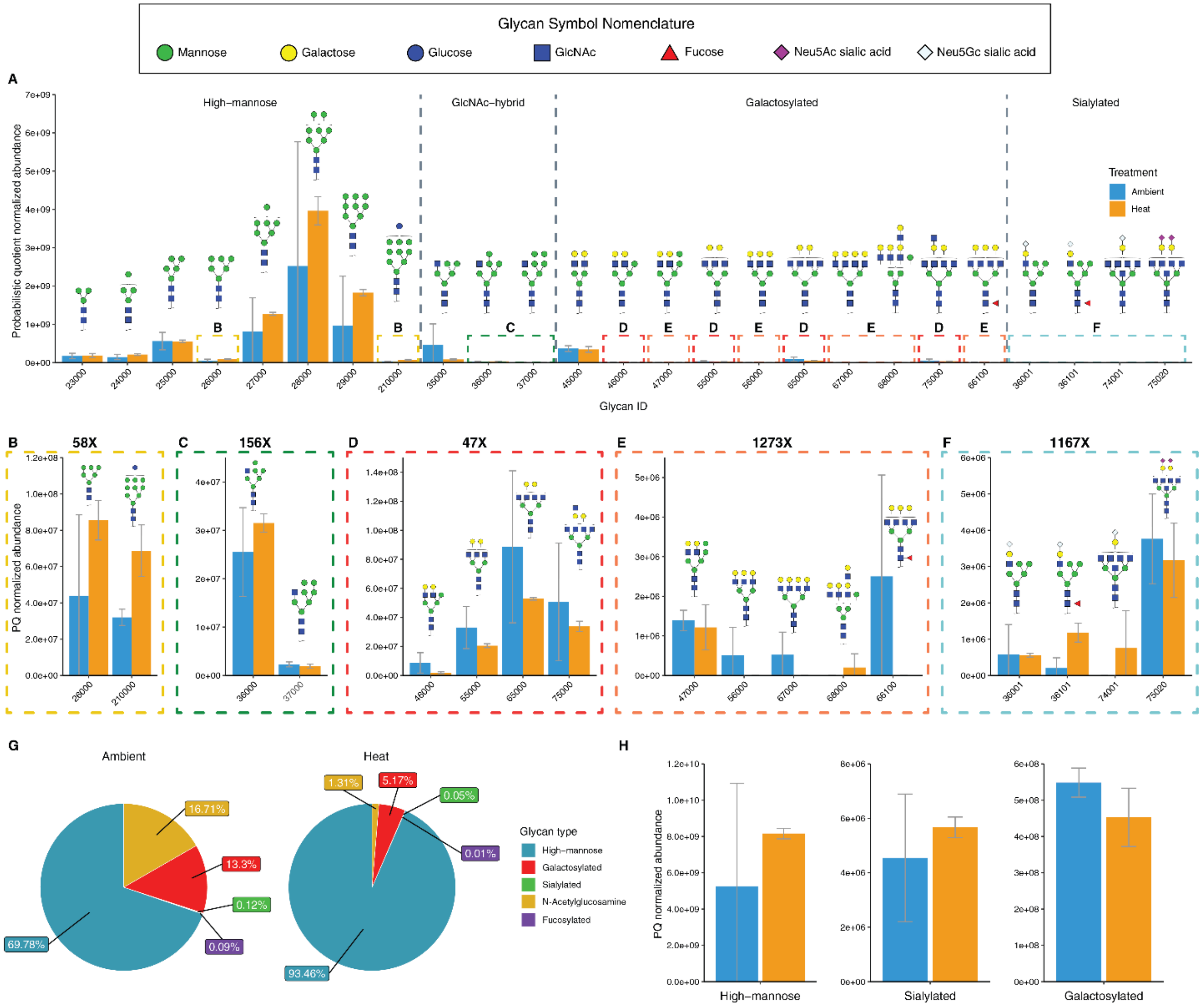

Supplementary Figure 3. Abundance of glycans from *Breviolum minutum* crude extract. **(A)** Probabilistic quotient normalized abundance of individual glycans separated into different categories based on their terminal residues. Boxed sections **(B)-(D)** are zoomed in graphs of corresponding boxes in **(A)**. **(G)** The relative abundance of glycans separated by type. "Fucosylated" category was separated from "Sialylated" and "Galactosylated" glycan groups. **(H)** Probabilistic quotient normalized abundance of glycans separated by type.

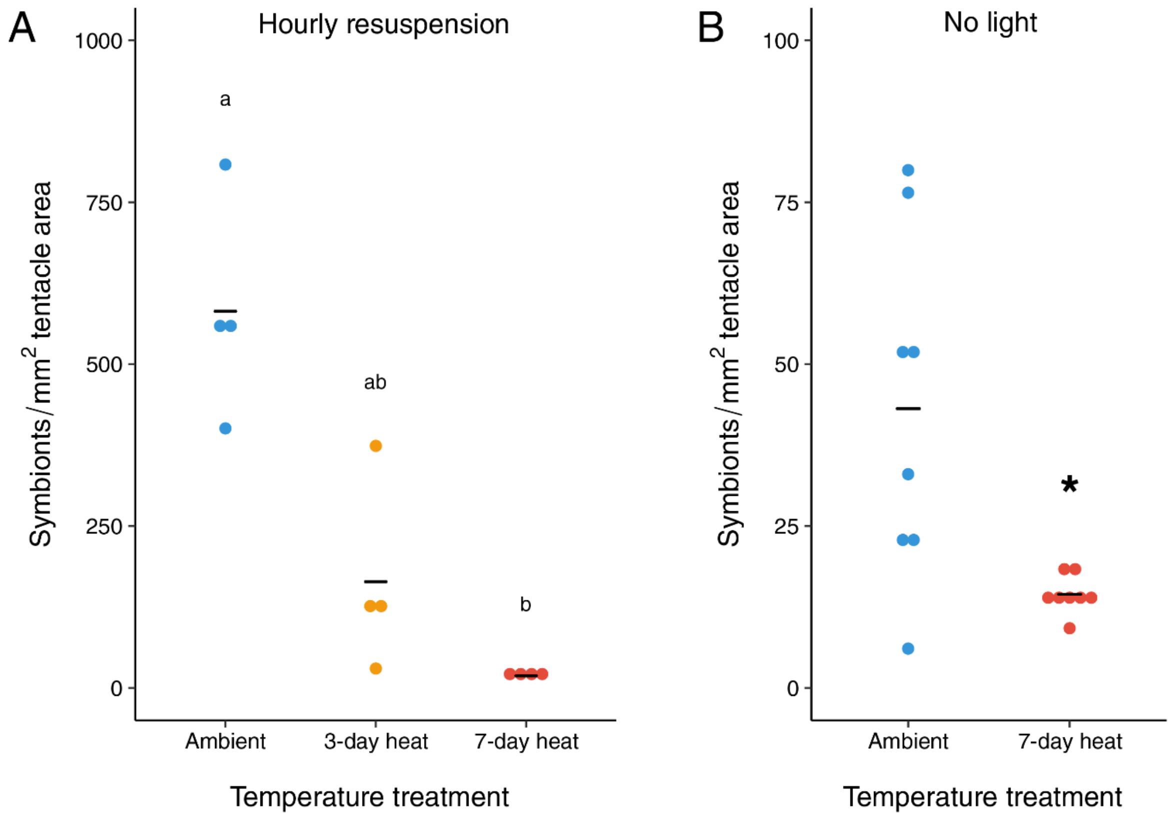

Supplementary Figure 4. Colonization with hourly resuspension or done in the darkness still caused a heat-stress associated decline in colonization. **(A)** Algae were resuspended every hour during the light period of inoculation to control for the effect of heat stress on algal motility. **(B)** Inoculations were conducted in the dark to minimize the effect of light-induced oxidative stress that would result from heat stressing the algae, but heat still reduced colonization. 3 day heat stressed algae were not included in this experiment. Letters indicate statistically significant differences ($p \leq 0.05$) as determined by Kruskal-Wallis and post-hoc Dunn Tests. Stars indicate significant differences ($p \leq 0.05$) as determined by Welch's t-test.

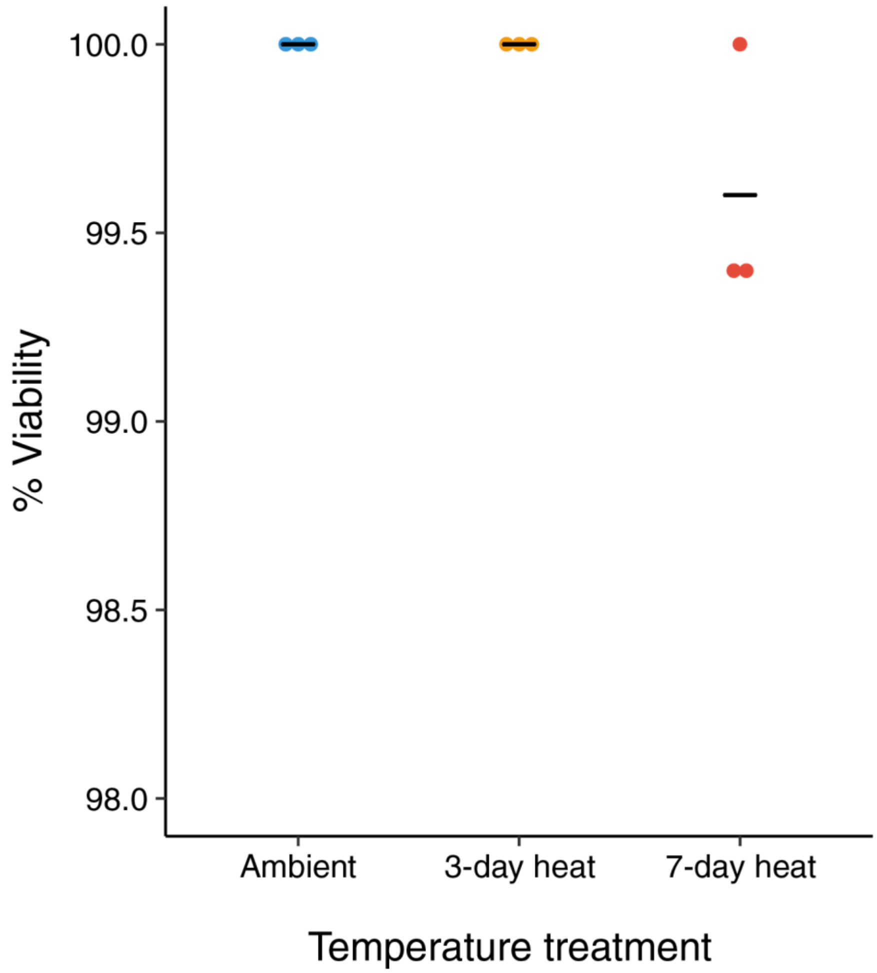

Supplementary Figure 5. Algae were stained with Evans Blue dye after heat stress to test cell viability. Heat stress caused a very small decline in cell viability that was not statistically significant.

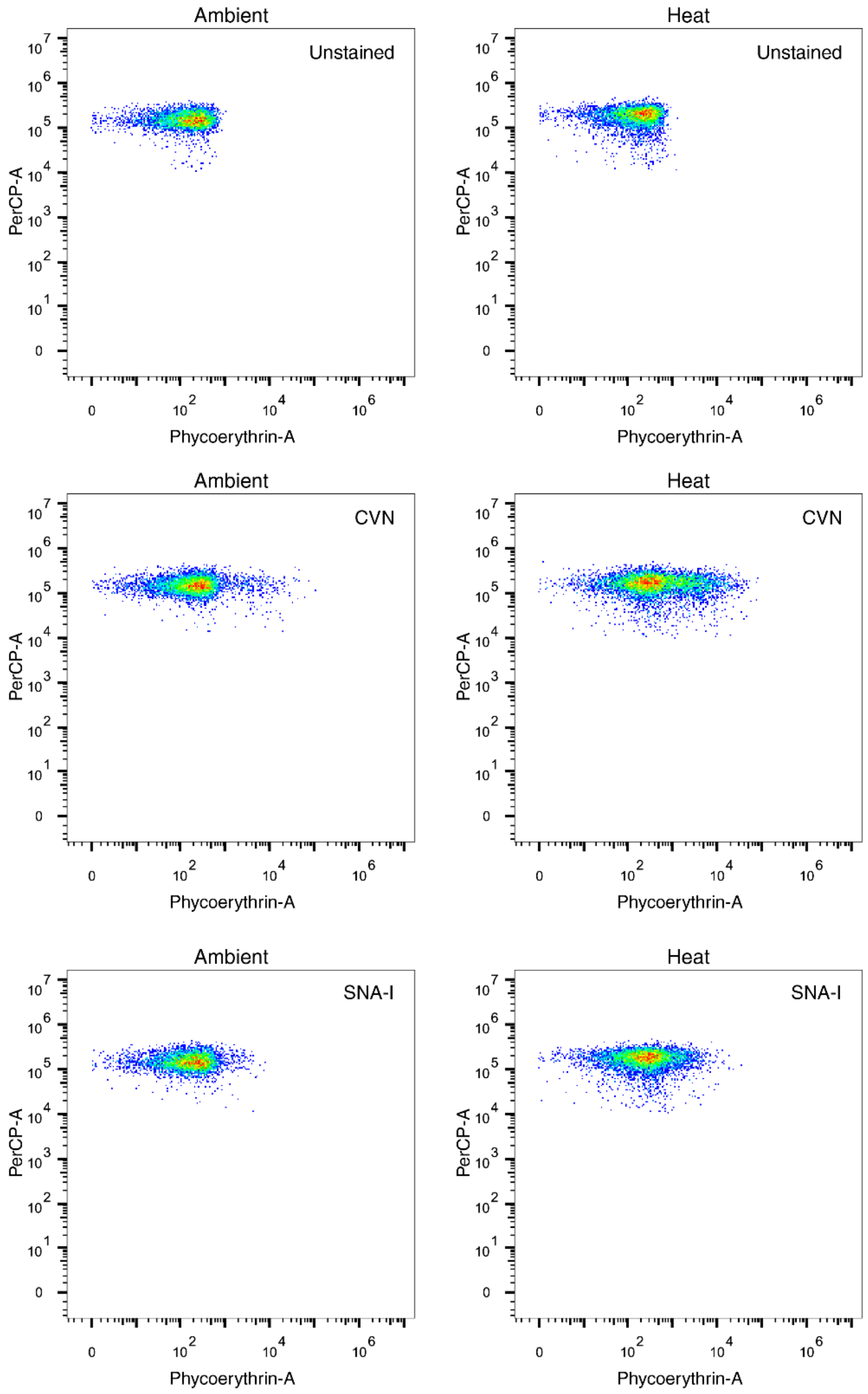

Supplementary Figure 6. Representative flow cytograms from one replicate each. The PerCP channel detected chlorophyll fluorescence and was used to gate live cells. The phycoerythrin channel detected the PE-labeled lectins, CVN and SNA-I. Unstained cells were used as blank controls and MFI values obtained from lectin labeled cells were corrected with MFI from the unstained cells.

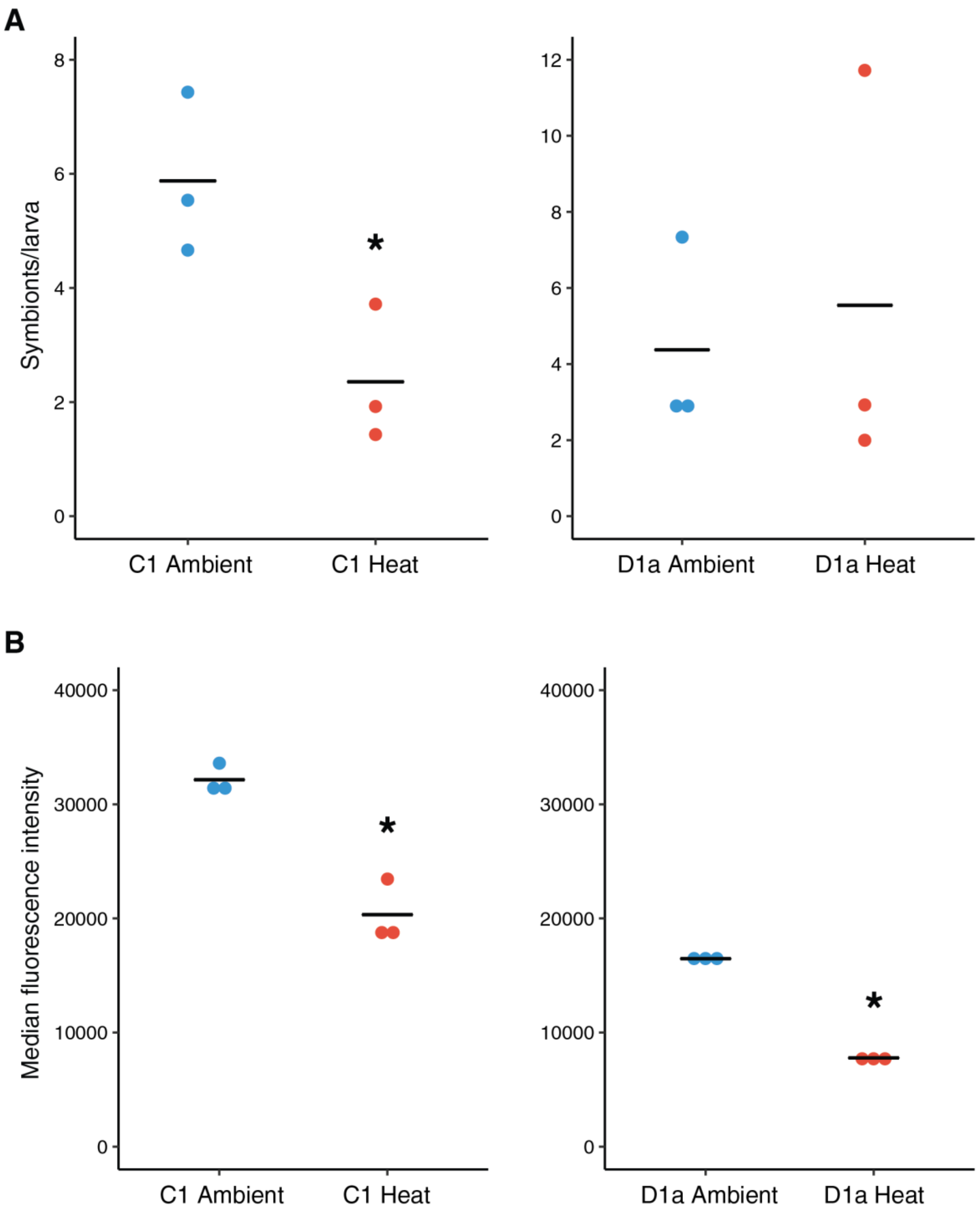

Supplementary Figure 7. **(A)** *Acropora tenuis* larvae were inoculated with ambient and heat stressed cultures of *Cladocoopium goreaui* (C1) or *Durusdinium trenchii* (D1a). Heat stress caused a decline in colonization in *C. goreaui* but not in *D. trenchii*. **(B)** Paraformaldehyde fixed cells were stained with PE conjugated CVN lectin and fluorescence detected with flow cytometry. Heat caused a decline in CVN labeling in both *C. goreaui* and *D. trenchii*. Stars indicate statistical differences ($p \leq 0.05$) as determined by Student's t-tests.
